# Laminin – myCAF interplay promotes α6-integrin-mediated breast cancer cell invasion

**DOI:** 10.1039/d5lc00711a

**Published:** 2026-08-21

**Authors:** María Virumbrales-Muñoz, Shelby A. Fertal, Karina M. Lugo-Cintrón, Jack R. Loken, Yalmarie Numan-Vazquez, Brian M. Burkel, Jing Zhang, Christian Franck, José M. Ayuso, David J. Beebe, Suzanne M. Ponik

**Affiliations:** a Department of Pathology and Laboratory Medicine, University of Wisconsin-Madison USA; b Department of Obstetrics and Gynecology, University of Wisconsin-Madison USA; c Carbone Cancer Center, University of Wisconsin-Madison USA ponik@wisc.edu; d Department of Cell and Regenerative Biology, University of Wisconsin-Madison USA; e Department of Mechanical Engineering, University of Wisconsin-Madison USA; f Department of Biomedical Engineering, University of Wisconsin-Madison USA; g Department of Dermatology, University of Wisconsin-Madison USA

## Abstract

It is well established that the tumor microenvironment (TME) plays a dynamic role in breast cancer progression. Stromal components of the TME, including fibroblasts and the extracellular matrix (ECM), provide biophysical and biochemical cues to drive tumor cell proliferation and invasion. However, how the interplay between activated fibroblast (CAF) subtypes and ECM composition impinges on breast cancer cell behavior is still not well understood. To address this gap, we leveraged microfluidic technologies to engineer a model of the breast TME that incorporates tumor cells, CAFs, and ECM components. Our model consists of a tubular duct filled with MDA-MB-231 or MCF-7 cells surrounded by a 3D collagen type I matrix with or without two human fibroblasts/CAFs. To further investigate how ECM composition affects the stromal compartment, we altered the matrix to include laminin-111 (LN) and thrombospondin-1 (TSP-1), which we previously found to be dysregulated in invasive ductal carcinoma compared to normal breast tissue. By incorporating LN or TSP-1 into the collagen matrix, we identified significant differences in collagen fiber metrics (fiber length, density, and width), matrix stiffness, and alterations in matrix remodeling by CAFs. Specifically, remodeling was more pronounced in LN- *vs.* TSP-1-enriched microenvironments populated by myofibroblast-like (myCAF-like) cells compared to inflammatory CAFs (iCAF-like). Notably, MDA-MB-231 cell proliferation and invasion were significantly enhanced under these same conditions, and invasion was dependent on the LN-binding receptor, α6-integrin.

## Abstract 2

Altering collagen composition with TSP-1 or LN changes fiber architecture and stiffness. In these matrices, myCAFs, but not iCAFs, enhance remodeling, and in LN-rich conditions, drive α6-integrin-dependent breast cancer invasion.

## Graphical abstract

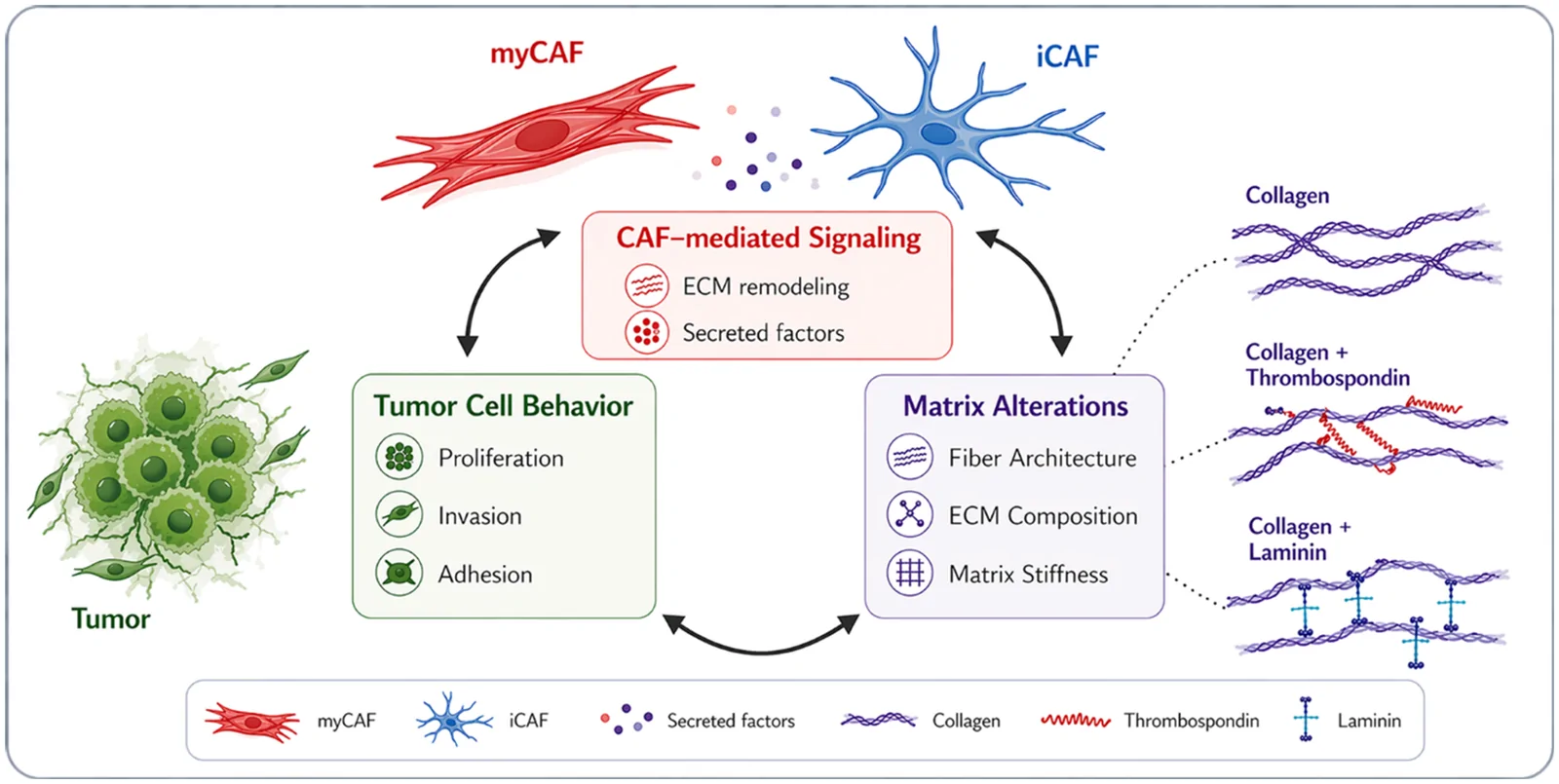

## Introduction

While early detection and surgery have improved outcomes for breast cancer patients, 30% of survivors will eventually progress to metastasis.^1^ Breast cancer cell invasion into the surrounding tissue is a key step in cancer progression that precedes metastatic disease. The invasive potential of breast cancer cells is highly influenced by the local tumor microenvironment (TME) that is composed of diverse cell types (*e.g.* fibroblasts, immune cells) and non-cellular components (*e.g.*, extracellular matrix). Together, these components dynamically interact to regulate the metastatic progression of breast cancer.^2^ Therefore, understanding how the interplay between specific components of the TME mechanistically regulates local invasion may reveal new targets for the therapeutic interception of this early step in metastasis.

A known factor within the TME that contributes to breast cancer pathogenesis and progression is the dysregulation of matricellular components. Mounting evidence has illustrated that the extracellular matrix (ECM), which serves as scaffolding for tissues, also plays a regulatory role in many hallmarks of cancer, including tumor cell differentiation, growth, survival, migration, and metastasis.^3,4^ Specifically, our prior proteomic analyses of the ECM revealed that the architecture and protein composition of invasive ductal carcinoma (IDC) are dysregulated compared to that of normal breast.^5^ Among the dysregulated proteins, we found several proteins from the laminin family of basement membrane proteins to be downregulated in IDC. Despite being less abundant in the TME, laminin-111 (LN) is one of many glycoproteins of the laminin family that have been linked to cell adhesion, differentiation, proliferation, protease secretion, and cancer cell metastasis.^6,7^ This is likely due to disruption of the basement membrane structure, leading to aberrant localization of LN in the tumor microenvironment. Indeed, several LN isoforms, along with the LN-binding integrin, α6β4, are upregulated at the tumor stromal interface of breast tumors.^8^ Conversely, thrombospondin-1 (TSP-1) was upregulated in IDC compared to normal tissue. TSP-1 is a matrix glycoprotein secreted by multiple cell types, including fibroblasts, that can promote fibrosis and inhibit angiogenesis. Interestingly, TSP-1 has been implicated in facilitating both tumor promotion and tumor inhibition in the TME. While some reports indicate that TSP-1 overexpression or administration inhibits tumor formation and progression,^9,10^ other models reported that TSP-1 promotes tumor occurrence and metastasis.^11^ Considering the conflicting information regarding LN and TSP-1 in the TME, there is a clear need to mechanistically study the effects of these individual ECM proteins in shaping the TME and identify their potential to promote or inhibit breast cancer cell invasion, leading to metastasis.

Among the many supporting cell types playing a role in the TME, cancer-associated fibroblasts (CAFs) are among the most abundant.^12^ CAFs are highly heterogeneous and well known to interact with and remodel the surrounding ECM.^13^ Recent studies have classified subtypes of CAFs based on their expression profiles, function, and spatial localization within the TME. Among these subtypes, myofibroblast-like CAFs (myCAFs) are characterized by their extensive matrix remodeling, while inflammatory CAFs (iCAFs) are known for the secretion of signaling molecules (including IL-6). While iCAFs are described to have tumor-promoting capabilities, there are reports linking myCAF with both promotion and inhibition of cancer cell invasion.^14,15^ Overall, CAFs are known to remodel the ECM, regulate immunosuppressive signaling, and impact therapy response. However, the functional contributions of specific CAF subtypes and how their interplay with different ECM proteins promotes breast cancer cell invasion have not been fully elucidated.

Arguably, the difficulty in dissecting the specific contributions of each of these TME components arises from a lack of models that allow combinatorial screening of these components and tracking of their functional effects in real time.^16^*In vitro* models have traditionally been quite useful in characterizing each individual component in a 2D setting. However, it is well-known that biophysical and biochemical cues from the 3D microenvironment drastically alter cell behavior, especially cell migration and proliferation rates.^17,18^ Conversely, *in vivo* models provide essential information regarding tumor progression in the context of complex interactions between multiple cell types and the ECM. A limitation of *in vivo* models is the lack of control of the TME. 3D *in vitro* models present an appealing alternative to bridge this important gap between traditional *in vitro* and *in vivo* models. 3D *in vitro* models of the TME provide the best of both worlds: lower cost and operative times, increased complexity (*e.g.*, 3D organization, potential integration of several cell types), and translatability to *in vivo* observations.^19^ The integration of many different components and physiologically relevant geometries within *in vitro* 3D models is not trivial.^20^ Therefore, microfluidic models represent a useful tool to generate complex microenvironmental conditions *in vitro* and offer a high degree of control and real-time inspection capabilities.^21,22^ We previously applied a microfluidic model to elucidate the effects of fibronectin-enriched matrices in modulating CAF effects in breast cancer progression.^23^ Here, we seek to understand the interactions between CAF phenotypes (subtypes) and other dysregulated ECM proteins in the breast TME to better understand the stromal impact on breast cancer cell invasion.

In this study, we systematically develop a customizable microfluidic model to investigate the complexity of the TME of IDC. We evaluated structural changes in the fibrous collagen matrix due to the addition of LN or TSP-1.^5^ Based on published phenotype, function, and secretory profiles,^12^ we evaluated the effects of two subtypes of mammary fibroblasts (iCAF-like or myCAF-like fibroblasts) on matrix remodeling and the impact of the resulting TME on cell invasion and proliferation in two breast cancer cell lines, triple-negative MDA-MB-231 and estrogen receptor-positive (ER+) MCF-7 cells. We identified increased invasiveness and proliferation of MDA-MB-231 breast cancer cells in the LN-enriched, myCAF-like microenvironment. Interestingly, knockdown of the laminin-binding receptor, α6-integrin, in MDA-MB-231 cells inhibited cell invasion. Together, these data demonstrate that the interplay between myCAFs and LN promotes an invasive TME that involves cancer cell adhesion and invasion through α6-integrin. Overall, this study illuminates our understanding of the interplay between ECM composition and CAF behavior in breast cancer progression. These results and future work in this model have the potential to aid in the development of novel therapeutic targets to detect and prevent metastasis, thereby improving the overall survival and quality of life of breast cancer patients.

## Results

### Characterization of enriched matrices in a microphysiological model

**Fig. 1:**
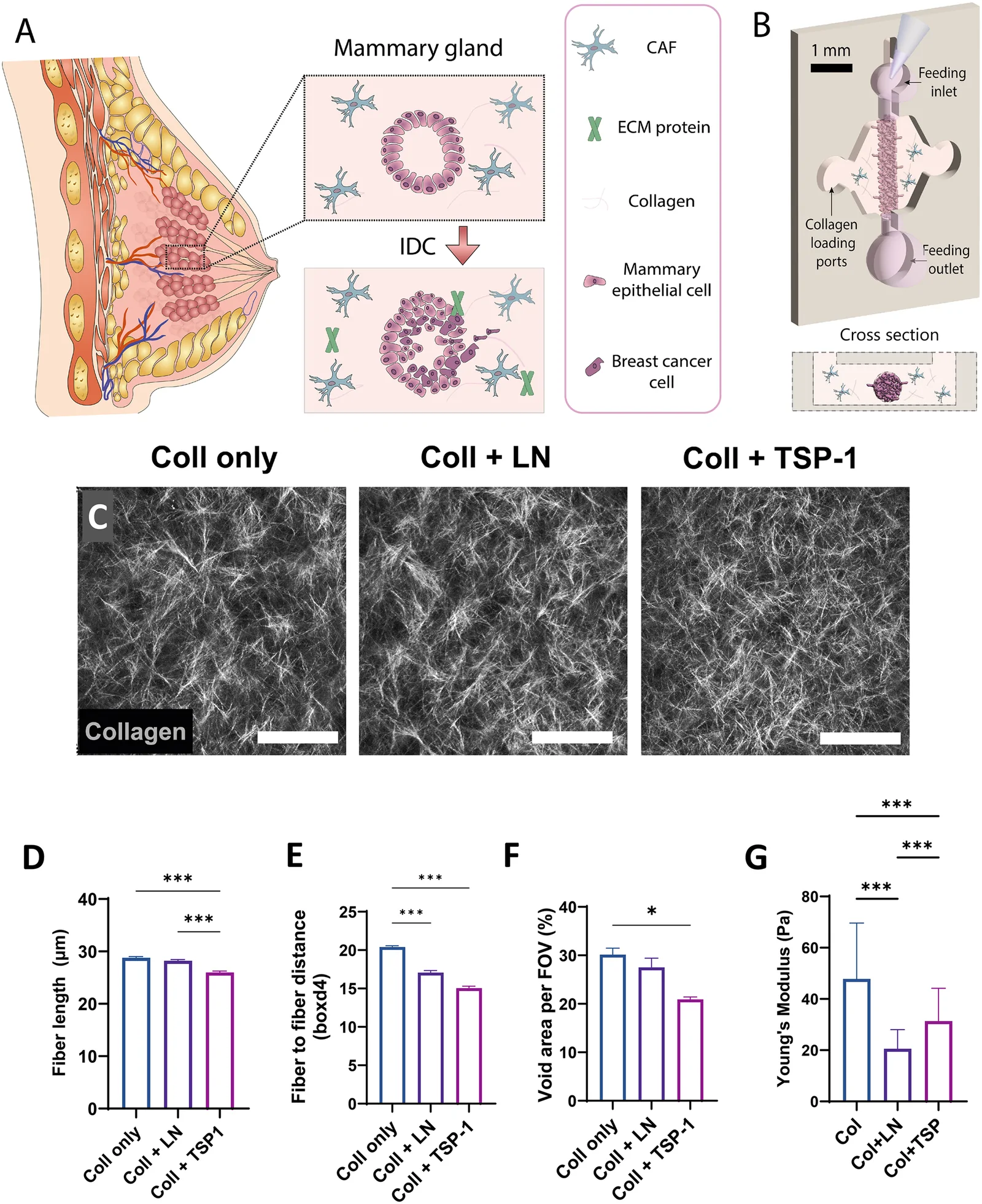
Inclusion of laminin (LN) and thrombospondin-1 (TSP-1) in hydrogels affects collagen (Coll) architecture. A) Schematic of breast cancer physiology, including an enlarged view of the epithelial duct or invasive lesion (invasive ductal carcinoma or IDC) with surrounding stroma and CAFs. B) Schematic of microfluidic model developed, which mimics breast cancer invasion into the surrounding matrix. C) Representative collagen second harmonic generation (SHG) imaging of control (Coll only) and ECM-enriched collagen matrices (Coll + LN and Coll + TSP-1). Scale bar = 50 μm. D–F) Quantification of collagen fiber metrics in each matrix condition revealed changes in fiber length (D), fiber density (E), and void area (black, non-fiber pixels between fibers) (F). Coll = collagen, LN = laminin-111, TSP-1 = thrombospondin-1. *N* = 3 with ≥2 technical replicates each. * = *p* ≤ 0.05, *** = *p* ≤ 0.001 *via* one-way ANOVA with Dunnett *post hoc* test. G) AFM was used to quantify alterations in stiffness due to matrix composition within a set of microfluidic devices. *N* = 3 with a minimum of 30 measurements for each condition. *** = *p* < 0.001 *via* one-way ANOVA with Kruskal–Wallis test.

Here, we generate a microphysiological model of the IDC microenvironment to evaluate the effects of specific ECM proteins and mammary fibroblast/CAF phenotypes on breast cancer progression. We based our model on an established microfluidic device engineered to create a hollow tubular structure embedded within a type-1 collagen (Coll) hydrogel (Fig. 1A and B).^23,24^ Within this device, the composition of the hydrogel was modified with ECM proteins and/or CAFs. In addition, the tubular structure can be filled with breast cancer cells, triple-negative MDA-MB-231 (MDAs) or ER+ MCF-7, to model a primary breast tumor surrounded by stromal matrix (Fig. 1B).

To characterize this model, we assessed the changes to the microenvironment after the stepwise addition of each new component. First, we incorporated two ECM proteins that we identified as dysregulated in breast tumors compared to healthy breast tissue,^5^ but whose specific roles in the transition to IDC are not fully defined. Particularly, we chose to evaluate the roles of LN and TSP-1. To study their contributions to breast cancer progression, we first asked whether the addition of each of these proteins stably incorporated into collagen type-I (Coll) hydrogels. TSP-1 has been shown to directly interact with Coll.^25^ In contrast, LN does not directly interact with Coll, but rather indirectly scaffolds to fibrillar Coll through other ECM proteins such as collagen-IV.^26,27^ In our model, the Coll hydrogel mixture is supplemented with Matrigel, which is comprised of LN, collagen-IV, and entactin proteins that can bridge LN–Coll interactions. Thus, we anticipate that the incorporation of TSP-1 and LN may potentially impact the structure and architecture of the collagen fiber network.

To begin characterizing TSP-1 and LN-enriched collagen matrices, we rinsed the hydrogels to remove any non-incorporated proteins before processing for Western blot analysis. We detected the presence of TSP-1 in the Coll + TSP-1 gel and an increase in LN in the Coll + LN hydrogel (Fig. S1). As anticipated, we detected low levels of LN in the Coll control hydrogel (Coll alone) due to the incorporation of Matrigel in the hydrogel mixture. This data supports that LN and TSP-1 were retained within the collagen hydrogels. Next, we evaluated how the inclusion of these ECM proteins influenced collagen fiber architecture. Using second harmonic generation (SHG) imaging of collagen, we visualized collagen fibers (Fig. 1C) and quantified structural changes in fiber architecture with CT-Fire and CurveAlign^28,29^ (Fig. 1D–F). We found that TSP-1 had a larger effect on fiber length than LN compared to Coll alone (9.7% decrease *vs.* 2.0% decrease) (Fig. 1D). To evaluate the fiber density of the matrices, we quantified changes in fiber-to-fiber distance and non-fiber area (void area). In CurveAlign, Boxd4 is a parameter that evaluates the distance of one fiber to the nearest 4 fibers, while void area is a global parameter that assesses the area within a field of view that is absent of fibers. Using these parameters, we found that Coll + TSP-1 and Coll + LN produced a denser structure (reduced distance to nearest fibers, boxd4) than Coll alone (Fig. 1E). Likewise, we found that the void area (black background) was significantly lower in Coll + TSP-1 (∼20%) compared to Coll alone (∼30%) (Fig. 1F).

Due to the identification of alterations in fiber metrics, we asked whether altering matrix composition would alter the stiffness of the hydrogel. To test this, we conducted atomic force microscopy (AFM) to quantify the stiffness (Young's modulus) across different matrix conditions. The stiffness of LN-enriched matrices was significantly lower than that of the TSP-1-enriched matrix. Furthermore, both LN and TSP-1-enriched collagen gels were significantly decreased in stiffness compared to Coll only (Fig. 1G). The relative differences in stiffness between matrix compositions are intriguing because our lab and others have demonstrated that differences in fiber architecture and matrix stiffness regulate stromal and tumor cell behavior.^30–32^

### Phenotypes and functional ECM remodeling of donor fibroblasts in TSP-1 and LN-enriched matrices

**Fig. 2:**
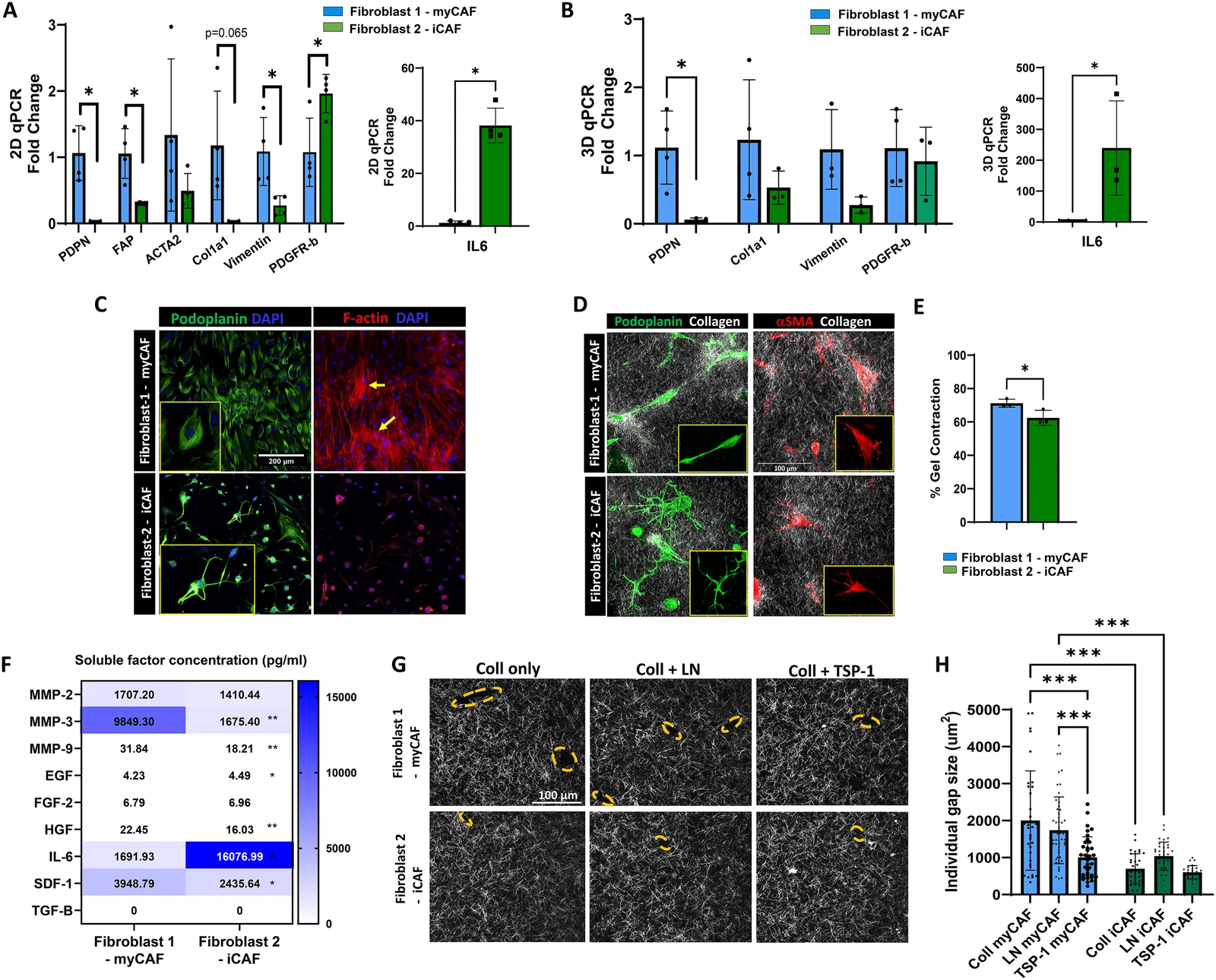
Characterization of two donor fibroblast phenotypes and their functional interactions with different 3D ECM compositions. A) Donor fibroblasts-1/myCAF-like & fibroblast-2/iCAF-like cells have differential transcriptional profiles of fibroblast activation markers when cultured in 2D (A) and 3D (B). C) Podoplanin immunofluorescence (IF) and phalloidin staining showed phenotypical differences in the 2D morphology between the two mammary fibroblast populations. Enlarged insets show spatial localization of podoplanin in fibroblast-1/myCAF-like *vs.* fibroblast-2/iCAF-like cells. Arrows depict F-actin rich stress fibers in the fibroblast-2/myCAFs. D) Representative IF images of podoplanin and αSMA in fibroblast-1 and -2 cells cultured in 3D collagen gels. Collagen SHG is visualized in grey scale. Insets show single channel IF for each protein and cell type. E) Quantification of collagen gel contraction by fibroblast-1/myCAF-like and fibroblast-2/iCAF-like. F) Quantification of secreted factors from fibroblast-1/myCAF-like and fibroblast-2/iCAF-like cells when cultured in Coll-only hydrogels. G) Representative SHG imaging of Coll and LN or TSP-1-enriched matrices after 3 days in culture with fibroblast-1/myCAF-like cells or fibroblast-2/iCAF-like cells. H) SHG image analysis reveals cell remodeled gaps in the matrix (indicated by dashed yellow regions) revealed the high remodeling activity of myCAF-like cells is modulated by ECM composition, with Coll + TSP-1 showing one of the lowest values for individual gap size. Coll = collagen, LN = laminin-111, TSP-1 = thrombospondin-1. (A, B, E, and F: *N* = 4 cultures per cell type, * = *p* < 0.05, Student's *t*-test comparison of fibroblast-1 *vs.* fibroblast-2) (H: *N* = 3 cultures per condition, *** = *p* < 0.001, one-way ANOVA with Kruskal–Wallis test).

To investigate the interplay between mammary fibroblasts and ECM-enriched Coll matrices, we used fibroblasts from two different donors (fibroblast-1 and fibroblast-2). Initially, we characterized the fibroblast phenotypes by assessing transcriptional profiles, cell morphology, secreted factors, and matrix remodeling capabilities in 2D and 3D. We evaluated the transcriptional profile of donor fibroblasts cultured in 2D and in 3D collagen hydrogels using a panel of fibroblast activation markers (Fig. 2A and B). In 2D, we identified significant differences in the expression of PDPN (podoplanin), FAP (fibroblast activation protein), PDGFR-β (platelet-derived growth factor beta), VIM (vimentin), and IL6 (interleukin-6) (Fig. 2A). Fibroblast-1 displayed higher expression of these markers, along with trends toward increased ACTA2 (alpha-smooth muscle actin or αSMA) and Col1a1 (collagen-1α1) compared to fibroblast-2. The exceptions were PDGFR-β and IL6, which significantly increased in fibroblast-2 *vs.* fibroblast-1 cells. Similar trends in transcriptional profiling were identified in 3D culture (Fig. 2B). The fibroblast-1 cells had significantly higher expression of PDPN and trends toward increased Col1a1 and VIM compared to fibroblast-2 cells, while IL6 was significantly increased in fibroblast-2 (Fig. 2B).

To validate the protein expression of selected fibroblast activation markers and define morphology differences, we conducted immunofluorescence imaging of podoplanin and actin in 2D and 3D (Fig. 2C and D). We chose podoplanin as it is a fibroblast activation marker that regulates actomyosin contractility and cell protrusions,^33–35^ and is associated with tumor progression and poor prognosis.^36,37^ We also visualized αSMA or F-actin (phalloidin staining) as markers of cell contractility. In 2D culture, the morphology of fibroblast-1 cells was elongated and spread into a confluent monolayer, while in 3D collagen hydrogels, we visualized an increase in local collagen fibers surrounding fibroblast-1 cells, suggesting a contractile phenotype (Fig. 2C and D). In contrast, fibroblast-2 cells were predominantly spindle-shaped with long protrusions in both 2D and 3D culture. Podoplanin was visualized in cell protrusions in the fibroblast-2 cells, while appearing as filamentous structures in fibroblast-1 cells. In 2D culture, robust F-actin stress fibers were visualized in the fibroblast-1 cells, while F-actin staining in fibroblast-2 cells showed minimal actin stress fibers. In 3D culture, αSMA could be visualized in fibroblast-1 cells bundling into a few stress fibers, while fibroblast-2 cells had diffuse localization of αSMA in protrusive structures. In addition to assessing markers of contractility, we quantified fibroblast contraction by measuring the decrease in the diameter of the collagen hydrogel, as done previously.^38,39^ Fibroblast-1 cells contracted the hydrogel significantly more (∼70% contraction from the initial volume) compared to fibroblast-2 cells (∼60% contraction) (Fig. 2E).

Next, we characterized the secretory profile of the donor fibroblasts cultured in 3D collagen hydrogels. Using multiplex bead-based ELISA we assessed the secretion of MMPs and growth factors (Fig. 2F). The ELISA panel revealed that fibroblast-1 cells secreted a significantly higher concentration of matrix metalloproteinases, MMP-3 and MMP-9, whereas fibroblast-2 cells secreted significantly higher concentrations of IL-6 and EGF (epidermal growth factor) (Fig. 2F). The changes in secreted factors, along with differences in transcriptional profiles and cell morphology, suggest the donor fibroblasts have unique characteristics of activated fibroblast (*i.e.*, CAF) function.

After this initial molecular and morphologic characterization, we aimed to determine how each donor fibroblast remodeled the ECM in the microfluidic model (Fig. 2G and H). To assess matrix remodeling by the donor fibroblasts in response to changes in ECM composition, we assessed the physical gap size created by cellular contractility and remodeling. We hypothesize that increased fibroblast-generated contractility and local secretion of MMPs (*i.e.* local matrix degradation) observed in fibroblast-1 cells will increase the gap size and distance between fibers calculated for the entire field of view compared to fibroblast-2 cells. We found the gap sizes generated by fibroblast-1 cells to be qualitatively (Fig. 2G) and quantitatively (Fig. 2H) larger in Coll alone and LN + Coll matrix conditions compared to fibroblast-2 cells. This is consistent with higher secretion of MMP-3 and -9 and an increase in stress fiber formation in the fibroblast-1 cells (Fig. 2C–F). When examining the effect of TSP-1 on fibroblast-induced matrix remodeling, we found that matrix remodeling by fibroblast-1 cells was suppressed in the Coll + TSP-1 condition compared to Coll alone and to LN + Coll. Despite the decrease in matrix stiffness identified in LN + Coll and TSP-1 + Coll matrices (Fig. 1G), there was no change in matrix remodeling in any of the matrix conditions by the fibroblast-2 cells, indicated by no significant change in gap size (Fig. 2H). Additionally, we found no difference in LN-enriched matrix remodeling compared to Coll alone by either of the donor fibroblasts. These data demonstrate that the effect of TSP-1 and LN to decrease matrix stiffness does not result in increased fibroblast-based matrix remodeling, as anticipated. Rather, matrix composition has a more complex relationship to matrix stiffness and cell response. Overall, our results point toward two distinct phenotypes of activated fibroblasts: one with high MMP-3 and 9 secretion and increased contractility, indicating active matrix remodeling that is characteristic of a myCAF-like phenotype, and the other with higher secretion of the inflammatory cytokine, IL-6, and epidermal growth factor, EGF, which is consistent with an iCAF-like phenotype. Based on these results, the fibroblast-1 donor cells will be referred to as myCAF-like or myCAF phenotype, and the fibroblast-2 donor cells will be referred to as iCAF-like or iCAF phenotype (Fig. 2).

### Crosstalk between CAF phenotypes and tumor cells modulates the secretory profile in the microenvironment

**Fig. 3:**
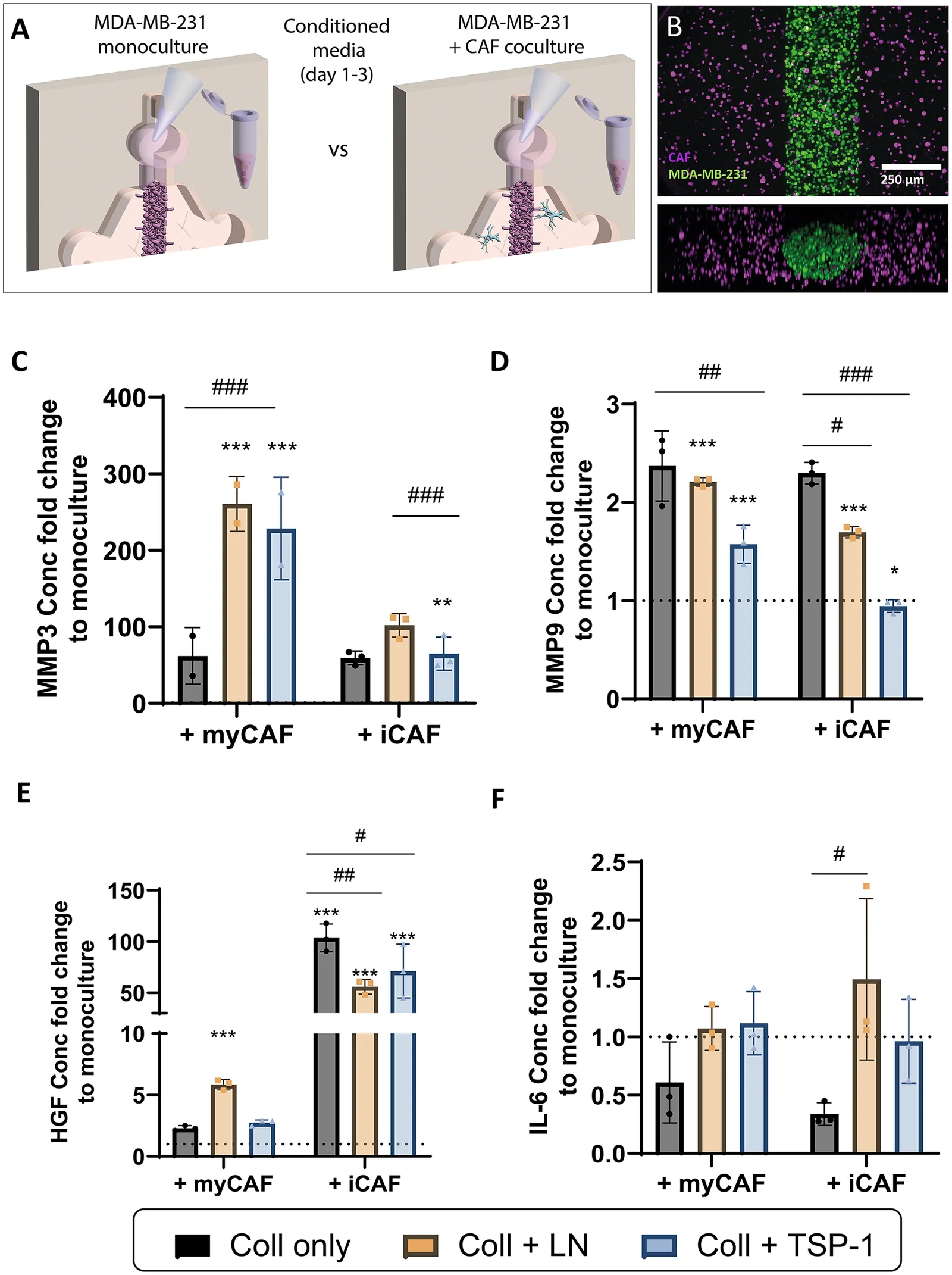
ECM composition modulates paracrine signaling from CAFs. A) Schematic of experimental design for evaluating CAF paracrine signaling in microfluidic IDC invasion model. B) For visualization of mammary fibroblasts (myCAF or iCAF phenotypes) labeled with CMTPX and MDA-MB-231 cells stably expressing GFP in the enriched collagen hydrogels. The representative image depicts myCAF-like cells (purple) and MDAs (green). C–F) Media from each culture was collected and quantified to revealed changes in the soluble concentrations of C) MMP3, D) MMP9, E) HGF, and F) IL-6 in our microfluidic model of IDC invasion. *N* = 3 with at least 3 pooled technical replicates each. Coll = collagen, LN = laminin-111, TSP-1 = thrombospondin-1. * = *p* ≤ 0.05, ** = *p* ≤ 0.01, *** = *p* ≤ 0.001 *via* two-way ANOVA + Tukey *post hoc* test with multiple comparison corrections. * = significant fold change differences to monoculture, # = comparisons are depicted between bracketed conditions.

Next, we aimed to verify that a differential secretory profile was maintained when using co-culture models (CAFs and tumor cells) with each different ECM composition (Fig. 3A). We analyzed the same soluble factors in MDA monocultures and co-cultures of MDAs + CAFs (myCAF or iCAF phenotypes) across all ECM conditions (Fig. 3A). A representative image of the co-culture model is visualized in Fig. 3B. We measured differences in the secretions of MMP-3, MMP-9, HGF, and IL-6 in the co-cultures normalized to monocultures (Fig. 3C–F). We found that MMP-3 secretion was significantly increased in myCAF-like co-cultures in LN- and TSP-1-enriched matrices (Fig. 3C). Specifically, myCAF phenotypic cell co-cultures produced a 260-fold increase in the secretion of MMP-3 in the LN-enriched matrix and a 228-fold increase when cultured in the TSP-1-enriched matrix compared to the MDA monocultures. MMP-3 secretion was also increased from iCAF-like co-culture *vs.* monoculture in the ECM-enriched models, but to a much lesser extent. MMP-3 secretion from iCAF-like co-cultures increased by 102-fold in LN and only 64-fold in TSP-1 over MDA monoculture (Fig. 3C). Both myCAF and iCAF phenotypic cells secreted similar levels of MMP-9 in all matrix conditions (Fig. 3D). MMP-9 secretion was the highest in the Coll alone matrix condition and lowest in TSP-1-enriched matrix for both CAF phenotypes. Despite MMP-3 secretion being higher in myCAFs than iCAFs, the iCAF-like co-cultures secrete higher amounts of HGF and IL-6 (Fig. 3E and F). Secreted HGF concentrations significantly increased in iCAF-like co-culture across all ECMs. Specifically, we find a 103-, 56-, and 71-fold increase in HGF in Coll, Coll + LN, and Coll + TSP-1, respectively (Fig. 3E). Conversely, myCAF-like cells only secrete a moderate amount of HGF in the LN-enriched condition (5.8-fold). Similar to HGF, IL-6 has been established as a key marker of the iCAF-like immunosuppressive phenotype.^37,38^ However, in our model, co-cultured iCAFs only increased secretion of IL-6 when cultured in LN-enriched matrices. iCAF co-cultures increased IL-6 concentrations by 1.49-fold in Coll + LN matrix compared to the Coll-only matrix condition (Fig. 3F). Notably, we did not detect a difference in IL-6 levels from myCAF-like cells in any of the matrix conditions. Together, these results demonstrate the specific interplay between CAF phenotypes and ECM composition to not only mediate matrix remodeling but also regulate the CAF/tumor secretome.

### Functional effect of CAF phenotype with ECM interactions on tumor cell proliferation and invasion

**Fig. 4:**
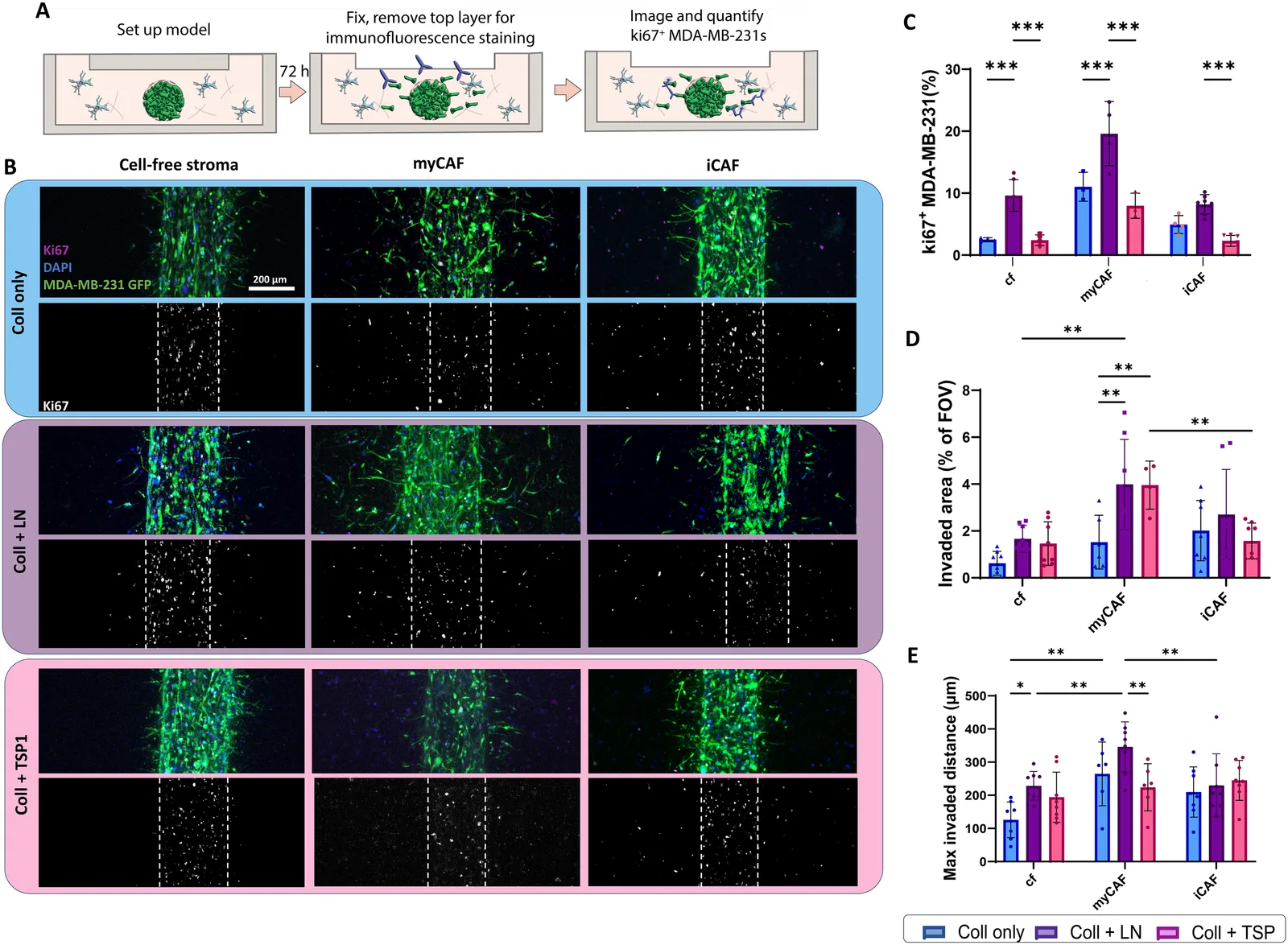
Stromal composition alters cancer cell proliferation and invasion. A) Schematic of experimental design for evaluating effects of stroma on tumor cell proliferation and invasion in microfluidic IDC invasion model. B) Representative confocal *z*-projected images of Ki67^+^/GFP^+^ tumor cell immunofluorescence in the models. Lower panels show Ki67^+^ tumor cells within and outside of the lumen (white dashed lines). C) Quantification of effects of stroma on the percentage of Ki67^+^ MDA-MB-231 cells in the microfluidic IDC invasion model. D) Area invaded by GFP^+^ MDA-MB-231 cells per field of view (FOV) and E) maximum invaded distance by GFP^+^ MDA-MB-231s after 3 days in stromal cell-free enriched matrices or with embedded CAFs (myCAF or iCAF phenotypes). Higher invasion in co-culture with myCAF-like cells in the Coll + LN enriched matrix was observed. *N* = 3 cultures per condition with at least 3 technical replicates each. Cf = cell-free, Coll = collagen, LN = laminin-111, TSP-1 = thrombospondin-1. * = *p* ≤ 0.05, ** = *p* ≤ 0.01, *** = *p* ≤ 0.001 *via* two-way ANOVA with Tukey *post hoc* test and multiple comparison correction.

To investigate the functional effect of the interplay between CAF phenotype and ECM composition on breast cancer cell proliferation, we used two breast cancer cell lines. We chose the invasive triple-negative cell line, MDAs, and the less invasive ER+ cell line, MCF-7s. Tumor cells were co-cultured with either myCAF-like or iCAF-like cells within all ECM compositions (Coll only, +LN, +TSP-1). To distinguish between cell types within the cultures, we used GFP-expressing MDAs or MCF-7s, and non-fluorescent CAFs (Fig. 4A). Under the clinically used label of progression, we quantified both proliferation and invasion (Fig. 4B). First, we evaluated the impact of CAF-mediated alterations in the TME on MDA proliferation by assessing Ki67 *via* immunofluorescence, as shown in representative images of myCAF-like and iCAF-like populated matrices (Fig. 4B). Image analysis was performed by generating masks of Ki67^+^ and GFP^+^ cells, which allowed for the quantification of MDA proliferation while eliminating Ki67^+^ GFP^−^ CAFs (Fig. S2A). Notably, the LN-enriched conditions showed an increase in MDA proliferation rates compared to both Coll-only and Coll + TSP-1 matrices for stromal cell-free, myCAF-like, and iCAF-like microenvironments (Fig. 4C). Under all ECM conditions, the myCAF-like populated microenvironments resulted in the highest MDA proliferation rates. Overall, the LN-enriched matrices with myCAF-like cells promoted the highest MDA proliferation rates. Unlike the MDAs, the MCF-7 cells did not change proliferation rates in response to any of the matrix conditions (Fig. S3A).

### myCAF interactions with LN-enriched matrix promote MDA-MB-231 invasion

To determine how tumor cell interplay with ECM composition impacts invasion, we quantified the total area and maximum distance of tumor cell invasion in each matrix condition. Invasion distances and areas were calculated outside the boundary of the lumen, which focused on tumor cells within the matrix after exiting the lumen (Fig. S2B). We found that modulating ECM composition alone was not sufficient to enhance MDA invasion area, as the cell-free stromal conditions averaged <2% invasion area regardless of ECM composition (Fig. 4D).

Next, we sought to determine how the interactions between CAFs and ECM composition altered tumor cell invasion. The myCAF-like populated matrices enhanced the area of MDA invasion (Fig. 4D) and promoted the greatest maximal distance of invasion (Fig. 4E). In both the Coll + LN and Coll + TSP-1 conditions, myCAF-like cells increased the overall area of MDA invasion compared to Coll only. The maximal distance of MDA invasion was significantly increased in the Coll + LN condition compared to Coll only and Coll + TSP-1. MDA invasion into iCAF-like populated microenvironments was not significantly altered in any ECM condition, despite the significant increase in HGF and IL-6 secretion in the iCAF-like co-cultures (Fig. 4D and E). Strikingly, very few MCF-7 cells invaded outside of the lumen boundary in any matrix condition (Fig. S3). Thus, only the maximum invasion distance was quantified. The MCF-7 cells invaded ∼10 μm into myCAF-like populated microenvironments, regardless of matrix composition (Fig. S3A). In the iCAF-like populated microenvironments, MCF-7 invasion ranged from 10–30 μm (Fig. S3B). In contrast, the MDAs invasion into the matrix ranged from 100–350 μm outside the lumen, depending on the stromal microenvironment conditions (Fig. 4D and E).

### Role of α6-integrin in MDA-MB-231 invasion in myCAF-populated, LN-enriched microenvironments

The interplay between myCAF-like cells and the Coll + LN enriched matrix promoted matrix remodeling (MMP-3 secretion), and the proliferation and invasion of MDAs. Focusing on this matrix combination, we hypothesized that MDA progression is mediated by the laminin-binding receptor α6-integrin (ITGA6). ITGA6 is a member of the integrin protein family, which mediates cell–ECM interactions. ITGA6 has been associated with increased cell proliferation, stemness, survival, migration, and resistance to therapy.^40–42^ ITGA6 forms heterodimers with integrins β1 and β4, both of which are highly specific laminin receptors.^43^ With this in mind, we tested the functional role of ITGA6 in MDA proliferation and invasion through siRNA-targeted knockdown.

**Fig. 5:**
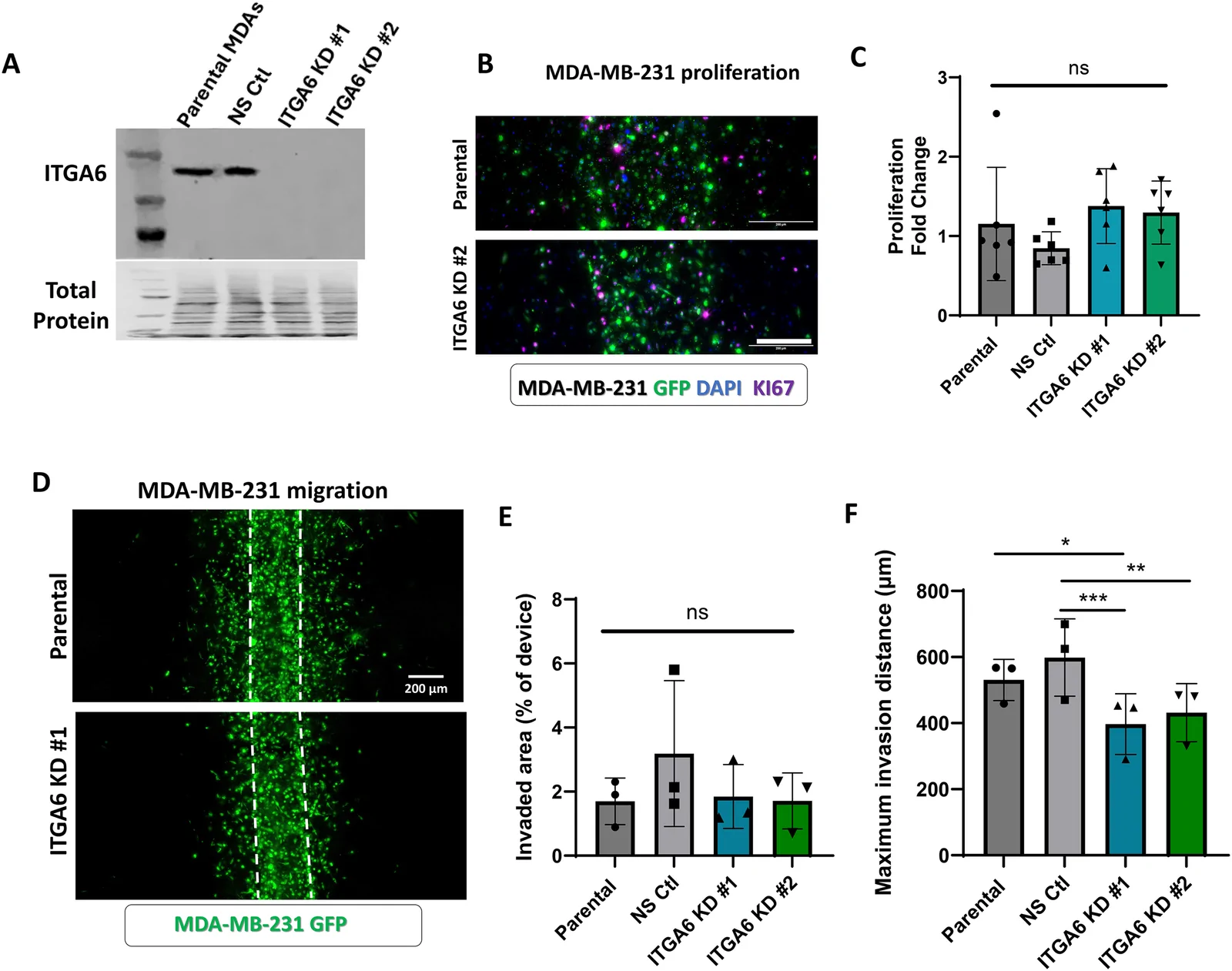
myCAFs promote breast cancer cell invasion in the LN-enrich microenvironment through an ITGA6-dependent process. A) MDA-MB-231 cells treated with siRNA for ITGA6 (ITGA KD #1 and #2). Knockdown of ITGA6 was confirmed by Western blot. B) MDA ITGA6 KD cells were cultured in a LN-enrich matrix with myCAFs. As controls, we used untransfected MDAs (parental) and non-targeted control MDAs (NS Ctl). Representative images of parental and KD #1 treated MDA stained for Ki67 after 72 h for parental and KD #2. Scale bar = 100 μm. C) Change in proliferation rates (Ki67^+^ MDAs) in control and ITGA6 KD MDAs. D) Representative fluorescent images of parental GFP^+^ MDA and KD #2 MDAs in the myCAF-like populated, LN-enriched microenvironment. E) Area invaded by cells per whole device, and F) maximum invasion distance by MDAs at 72 h. *N* = 6 cultures per condition, * = *p* ≤ 0.05, ** = *p* ≤ 0.01, *** = *p* ≤ 0.001 *via* one-way nested ANOVA with Tukey *post hoc* test and multiple comparison correction.

In this experiment, ITGA6 expression was knocked down (KD) in MDAs with two different siRNAs (KD #1 and KD #2). MDA expression of ITGA6 protein was compared between ITGA6 knockdown, non-targeted control siRNA (NS Ctl), and parental (untransfected) cells by Western blot (Fig. 5A). MDAs transfected with KD #1 and KD #2 had undetectable ITGA6 expression, whereas robust ITGA6 was detected in parental and NS control conditions. Control or KD MDAs were then seeded in the lumen of myCAF-populated Coll + LN matrices. After 72 h of culture, MDA invasion and proliferation were analyzed. KD of ITGA6 did not alter the percentage of proliferating MDAs (Fig. 5B and C). Ki67 staining revealed heterogeneity, but no statistical difference in the percentage of proliferative cells in either ITGA6 KD cell line compared to scrambled or NS control cells (Fig. 5C). This result suggests that proliferation in the LN-enriched matrix may be regulated through a different integrin receptor, such as α3β1. Next, we assessed the effects of ITGA6 KD on overall cancer cell invasion (Fig. 5D–F). We observed a statistically significant decrease in the maximum distance invaded between NS Ctl and both ITGA6 KD cell lines (Fig. 5D and F). However, a significant decrease in invasion distance was identified only in the ITGA6 KD #1 compared to parental MDAs. Interestingly, we did not observe differences in tumor cell-invasion area (Fig. 5E). These results indicate that ITGA6 facilitates MDA cancer cell invasion, specifically in the myCAF-populated, LN-enriched microenvironment.

## Discussion and conclusions

Aggressive invasion of breast cancer cells has been associated with pathological changes in the stromal breast tumor microenvironment.^7,41,44,45^*In vitro* studies aimed to investigate the mechanisms of 3D tumor cell invasion have primarily focused on breast cancer cell response to modulating collagen fiber architecture and/or collagen density,^30,32,46–48^ with limited mechanistic studies including the contribution of CAFs.^23,49–51^ Moreover, the concept of CAF subtypes regulating specific aspects of breast cancer invasion is relatively new,^52^ and few studies have definitively characterized how these different subtypes/phenotypes functionally contribute to matrix remodeling that supports breast cancer invasion.^23,50,51,53^ Similarly, we are only beginning to understand how the complex composition of the breast tumor ECM influences CAF-mediated matrix remodeling and subsequent tumor cell behavior.^23,54,55^ Thus, *in vitro* culture platforms capable of modeling the heterogeneity of the tumor microenvironment, including stromal cell types and the ECM, are required to elucidate the complex mechanisms that regulate breast tumor cell invasion.

In this study, we leveraged a 3D microfluidic model to dissect the interplay between two CAF subtypes and two ECM components, namely laminin-111 (LN) and thrombospondin-1 (TSP-1), in remodeling the breast TME. Our findings reveal that LN and TSP-1 significantly alter the collagen fiber network and matrix stiffness. In line with the reported tumor-suppressive role of TSP-1,^9,10^ incorporation of TSP-1 into the Coll matrix reduced ECM remodeling by both CAF phenotypes (iCAFs and myCAFs). The Coll alone and LN-enriched matrices promoted myCAF-mediated remodeling (contraction-induced gaps in the matrix) compared to the TSP-1 + Coll matrix. We also made the novel finding that the LN-enriched, myCAF-populated microenvironment, which had the softest matrix, specifically enhanced maximal MDA tumor cell invasion. This result indicates that the biophysical cues in the tumor microenvironment driving tumor cell invasion involve complex interactions between ECM composition and CAF subtypes. However, neither CAF subtype nor matrix composition was sufficient to enhance the proliferation or invasion of MCF-7 cells, which have a low invasive phenotype. These results demonstrate that the dynamic interactions between fibroblasts and ECM result in heterogeneous biophysical cues and substrate-specific tumor cell adhesions that drive the invasion of aggressive cancer cells.

While we and others have found LN to be downregulated in breast cancer tissue,^5,56^ our data point to a critical role for stromal LN to enhance myCAF-mediated matrix remodeling and tumor cell invasion. When the basement membrane is disrupted during the transition to invasive breast cancer, LN has been shown to be upregulated at the tumor stromal interface.^8^ In the context of the *in vivo* breast TME, the laminin-binding integrin, α6β4, is positively correlated with tumor grade in most instances, indicating that this integrin promotes tumor progression and metastatic spread. Conversely, there are cases where α6β4 is negatively correlated with tumor invasion and formation of metastases (reviewed in ref. 57). While the mechanisms for this discrepancy remain unclear, it has been shown that epithelial dissociation from the laminin-rich basement membrane leads to the relocalization of α6β4 to actin-rich filopodia and lamellae, where it plays a role in 2D cell migration.^58,59^ Further, α6β4 has been shown to regulate breast cancer cell migration and invasion through the activation of FAK^60^ and to promote Rho-mediated contractile ECM remodeling.^61^ However, these studies have not taken into account the interplay between α6β4-expressing tumor cells and CAFs within the 3D TME. Here, we show that the myCAF-LN remodeled microenvironment supports an increase in MDA proliferation and invasion. Our experiments reveal that α6-integrin mediates MDA cell invasive, but not proliferative, effects of the myCAF-LN remodeled microenvironment, as knockdown of α6-integrin significantly reduced the maximum invasion distance of MDA cells without affecting the overall invaded area. Thus, our data suggest a selective role for α6-integrin in aggressive breast cancer invasion and a potential functional regulator of metastatic spread, specifically when interacting with a myCAF remodeled, LN-enriched matrix. Future studies will test whether α6-mediated tumor cell invasion is unique to LN-111 or whether other α6-binding laminin isoforms (*e.g.* LN-332 or LN-511) promote the same response.

In our model, the changes in matrix composition led to alterations in collagen fiber architecture and matrix stiffness that impacted cell invasion in unexpected ways. Others have demonstrated that coarse fiber networks, similar to those we observed in the Coll matrix condition (Fig. 1C–F), have high stiffness. Along these lines, the addition of TSP-1 or LN decreased the coarseness of the matrix, resulting in significantly lower elastic moduli than the Coll only condition. According to a basic model of durotaxis, increased matrix stiffness is traditionally associated with enhanced cell invasion, but the LN + Coll hydrogels, the most invasive environment in our study, exhibited the lowest modulus. These findings shed light on the complexity of biophysical cues that regulate cell invasion in the heterogeneous tumor microenvironment. Our observed decoupling of micro-scale (cell-scale) and macro-scale (global stiffness) mechanics that occur with changes in matrix composition suggest that durotaxis, as defined by migration or invasion toward increased stiffness,^62^ does not fully account for the alterations in tumor cell invasion in our 3D model system. We propose that in a collagen fiber network, mechanical cues from the abundance and organization of collagen fibers (which provide topotaxis,^63^ or contact guidance^64^ cues for cell invasion) may serve as a foundational mechanical property of the TME that can be modulated by compositional changes in matrix proteins. Our studies reveal that biophysical cues can be further modulated by specific CAF phenotypes. CAFs are known to play a critical role in shaping the local biophysical features of the tumor microenvironment through the deposition of desmoplastic stroma, which has been correlated with breast cancer aggression.^65^ Surprisingly, targeting specific drivers of desmoplasia, such as CAFs, can either enhance or inhibit tumor progression.^52,66^ These conflicting effects suggest that the heterogeneity of CAF phenotype and the functional interplay with the fibrous 3D biophysical tumor microenvironment is not fully understood. Our data suggests that myCAF-like cells can modify biophysical cues in the matrix by generating contractile forces on the matrix through integrin-mediated adhesion, potentially driving tensotaxis (defined as migration or invasion toward higher tension).^67,68^ It is possible that the matrix remodeling myCAF phenotype contributes to spatial heterogeneity of mechanical cues by pulling on specific fibers and remodeling localized regions of ECM, which could create zones of high tension that the tumor cell senses, even if the rest of the hydrogel remains soft. Future studies are required to define the interplay between cell-scale biophysical alterations in the matrix that regulate tumor cell invasion. Overall, this data highlights the need to consider how all of these complex modulatory components within the tumor microenvironment contribute to the mechanical cues that regulate tumor cell invasion.

This study also underscores the functional importance of CAF–ECM interactions in shaping a pro-invasive microenvironment. More broadly, we highlight the power of 3D microfluidic models as reductionist systems that allow controlled manipulation and observation of tumor microenvironment (TME) components in real time. Our platform enables systematic testing of ECM proteins, stromal cell subtypes, and tumor cells within compartmentalized geometries that reflect physiologically relevant tissue organization.^20^ As demonstrated, this device readily incorporates primary mammary fibroblasts/CAFs and ECM proteins identified from invasive ductal carcinoma proteomics datasets,^5^ and can be expanded for investigation into the role of other tumor types, additional stromal cell populations (*e.g.*, macrophages, T cells), patient-derived samples, cytokine supplementation, or other dysregulated ECM components. These studies will be particularly important to identify the role of iCAF secreted factors, such as IL6 and HGF, on the tumor immune response and whether this microenvironment supports breast cancer progression.

In conclusion, our findings support the idea that interactions between CAF subtypes and ECM components critically influence breast cancer cell proliferation and invasion. Our microfluidic device offers a physiologically relevant yet reductionist platform to interrogate these complex interactions. Future studies using this human-relevant approach hold significant promise for translational applications, including the identification of novel therapeutic targets and the design of personalized treatment strategies for metastatic breast cancer.

## Methods

### Cell culture

Human mammary adenocarcinoma cells MDA-MB-231 (ATCC) and MCF-7 (ATCC) were stably transfected with turbo green fluorescent protein (GFP) to visualize and quantify cell invasion.^69^ Immortalized human mammary fibroblasts (fibroblast-1/myCAF-like) and carcinoma-associated fibroblasts (fibroblast-2/iCAF-like) derived from the stromal vascular fraction of a reduction mammoplasty or isolated from breast cancer biopsy tissues, respectively (a kind gift from the Kuperwasser lab, Tufts University).^70^ All cells were routinely cultured in high glucose DMEM (Gibco, 11965092) supplemented with 10% fetal bovine serum (FBS, VWR, 97068-085) and 1% penicillin/streptomycin (ThermoFisher, 15140-122) and were kept in a humidified incubator at 37 °C with 5% CO_2_. All cells were cultured to 90–95% confluency and with media changes every 2–3 days.

### Microdevice fabrication

LumeNEXT fabrication was performed as previously described.^23,24^ Briefly, the microdevice consists of two polydimethylsiloxane (PDMS) layers, that sandwich a suspended PDMS rod. High-density rat-tail collagen type 1 (Corning, 354249, referred to as Coll through the text) was diluted with 5× PBS (PBS, Fisher Scientific, BP3991, diluted with DI water) and neutralized with 0.5 M NaOH (Fisher Scientific, S318) to a final concentration 2.0 mg ml^−1^, and a pH of 7.4. This collagen mixture was diluted with fibrinogen (Sigma-Aldrich, F8630), Matrigel (Sigma Aldrich, CLS356234), and media to achieve a final concentration of 2.00 mg mL^−1^. EHS mouse laminin (also known as laminin-111, Corning, 354232) or human recombinant thrombospondin-1 (Millipore-Sigma, SRP4805) was added to final concentrations of 130 and 1.35 μg ml^−1^ in the collagen matrix, respectively. The stock and final concentrations used for this mixture are detailed in Table 1. Fibroblast-1/myCAF-like or fibroblast 2/iCAF-like cells were suspended in the hydrogel mix at a concentration of at 2500 cells per μl. After polymerization of a hydrogel within the device chamber, the rod is removed with tweezers to reveal a tubular lumen structure. A 1.5 mg ml^−1^ collagen master mix solution (collagen + fibrinogen + Matrigel ± LN or TSP-1) was prepared as indicated above, with the addition of the tumor cells at a final concentration of 16 666 cells per μl. Then, the media from the lumen was aspirated and 3 μl of the collagen master mix solution with tumor cells was loaded through the input port. Collagen was polymerized as described in previous sections.

Final reagent concentrations in the thrombospondin- and laminin-enriched collagen hydrogel mixtures

**Table:** 

	Stock used	Final concentration
Collagen	8.14 mg mL^−1^	2.00 mg mL^−1^
PBS	5×	1×
NaOH	0.5 M	5.25 mM
Fibrinogen	20 mg mL^−1^	0.71 mg mL^−1^
Matrigel	10 mg mL^−1^	0.177 mg mL^−1^

**Table:** 

+ one of the following ECM proteins		
Laminin	0.1 mg mL^−1^	130 μg mL^−1^
Thrombospondin	1.72 mg mL^−1^	1.35 μg mL^−1^

### Atomic force microscopy (AFM)

Matrix stiffness was measured using an MFP-3D-BIO atomic force microscope (Oxford Instruments – Asylum Research, Santa Barbara, CA, USA). All measurements were performed in 1× PBS at room temperature. Briefly, a 25 μm diameter polystyrene colloidal probe (NovaScan, USA) with a nominal spring constant of 0.12 N m^−1^ was used for indentation experiments. Prior to measurements, the cantilever spring constant was calibrated in PBS using the thermal noise method.^1^ The calibrated spring constant in liquid was 0.0775 N m^−1^ and this value was used for all calculations. Collagen hydrogels (Coll, Coll + TSP-1, and Coll + LN) were prepared as described above, with a final gel thickness was approximately 600 μm, minimizing substrate effects during indentation. Indentation was limited to ≤2 μm to remain within the small-strain regime relative to the probe radius (*R* = 12.5 μm) and to avoid nonlinear strain-stiffening effects. Force–distance curves were acquired at randomly selected surface locations, with 100 curves collected per group from two independently prepared samples. Curves with poor contact or excessive adhesion were excluded from analysis.

Young's modulus was determined by fitting the approach portion of the force–indentation curves to the Hertz model for a spherical indenter:
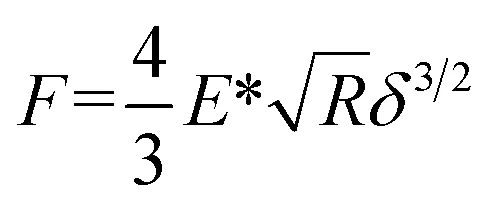
where *F* is force, *R* is the probe radius, and *δ* is indentation depth. The reduced modulus E* is related to Young's modulus *E* by:
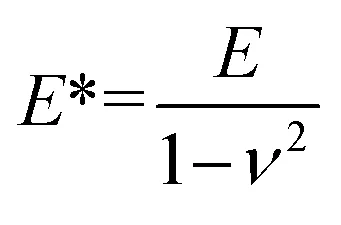
Force–indentation curves were acquired at an approach velocity of 5 μm s^−1^. At this loading rate and indentation depth, the measured response reflects an apparent Young's modulus that may include contributions from viscoelastic and poroelastic effects inherent to collagen hydrogels. Because all measurements were performed under identical conditions, the data represent an apparent modulus incorporating both elastic and time-dependent mechanical contributions, enabling robust relative comparisons across matrix compositions. Collagen hydrogels were assumed to be incompressible, and a Poisson's ratio of *ν* = 0.5 was used, consistent with commonly reported values for hydrated biopolymer networks. While deviations from this assumption may influence absolute modulus values, they are not expected to affect relative comparisons between conditions. Fitting was restricted to the initial 0.5–1.0 μm of indentation to minimize large-strain stiffening effects. A MATLAB-based AFM data processor, FRAME,^2^ was used for the fitting.

### qPCR

RNA was isolated from fibroblasts seeded in a 6 well plate (2D) or within a 3D collagen hydrogels (described above) using the RNEasy RNA extraction kit (Qiagen) and reverse transcription was done with the high-capacity RNA to cDNA kit (Thermo Fisher #4387406). RT-qPCR was conducted using Taqman Probes (Thermo Fisher) detailed in Table 2. RT-qPCR reactions were run using Lightcycler 480 probes master mix (Roche 04887301001) in Roche's Lightcycler 480 II (Roche Molecular Systems, Indianapolis, IN). Target gene expression was normalized using the geometric mean of the reference genes HPRT and RLBP0. Relative gene expression fold changes were determined using the 2^−ΔΔCt^ method compared to the reference genes.

**Table 2:** Taqman-style qPCR primers used to evaluate canonical CAF markers, and internal control primers

Gene name	Taqman catalog number
HPRT1	Hs02800695_m1
RLBP0	Hs99999902_m1
IL-6	Hs00174131_m1
PDPN	Hs00366766_ml
FAP	Hs00990791_m1
ACTA2	Hs00909449_m1
COL1A1	Hs00164004_m1
PDGFR-b	HS01019589_ml
VIM	Hs00958111_m1

### Cytokine secretion assay

Multiplexed protein secretion analysis was performed on culture media from devices using the Magnetic Bead-Based Multiplex ELISA system MAGPIX (Luminex Corporation) Human MMP Magnetic Panel (3-Plex) (R&D Systems, FCSTM07-03) and human cytokine panel bead kit (R&D Systems, LXSAHM-10) as described elsewhere.^71^ Collected media from 6 cultured vessels at 24 and 48 h were pooled to increase the sample volume in each cultured condition and processed as per the manufacturer's protocol. Data were collected with xPonent software (Luminex), and soluble factor concentrations in media were calculated using mean fluorescence intensities (MFI) by creating a standard curve for each analyte using a five-parameter logistic (5-PL) curve fit in GraphPad Prism.

### Image acquisition and image analysis

Collagen hydrogel structure was visualized by second harmonic (SHG) using a custom-built inverted multiphoton microscope (Bruker Fluorescence Microscopy, Middleton, WI), as described previously.^72^ The system consisted of a titanium:sapphire laser (Coherent), an inverted microscope (Nikon, Eclipse Ti, Melville, New York, NY, USA), and a Nikon Apo 40×/1.25 WI λS objective. Collagen fibers were excited using an 890 nm, an emission bandpass filter of 440/80 nm, and a GaAsP photomultiplier tube (H7422P-40, Hamamatsu). 3 single-plane images were collected per device (4 devices per condition) at a 100 μm distance from the bottom of the gel, to avoid edge effects. To capture 3D immunofluorescence, a 525/70 bandpass filter was added to the dichroic cube, and a *z*-stack with 3 μm steps were collected. To track cancer cell migration out of the lumen, a Leica SP8 3× STED confocal/super-resolution microscope (UW-Madison Optical Imaging Core) with a 10× objective or a Leica Thunder with a 20× objective. For each device, a *Z*-stack imaging was performed after 72 h in culture. Confocal image stacks (every 15 μm) were acquired after 72 h in culture, and then *Z*-projected, using ImageJ.^73^

Gap sizes from SHG images of the different collagen matrices were analyzed using ImageJ. Fiber characterization was done using CT-Fire and CurveAlign software (UW-Madison LOCI). Images were analyzed through both programs to determine fiber length, alignment, and fiber distance to next nearest 4 fibers (boxd4), as done previously.^5,28,29^ The invaded area was measured as the area outside of the lumen structure occupied by MDA-MB-321 cells was measured and presented as % of FOV. The maximum invasion distance was measured manually on both sides of the lumen structure, capturing the 10 furthest cells from the edge of the lumen. To quantify Ki-67 in MDA-MB-231 only, a mask was made using the GFP signal from MDA-MB-231 and was used to exclude DAPI signal from fibroblasts and Ki67^+^ fibroblasts. Then, automatic particle counting was done for both DAPI and Ki67 to calculate the percentage of Ki67^+^ MDA-MB-231s (Fig. S2).

### Fluorescent staining

Stock solutions of cell tracker red CMTPX (Thermo Fisher, C34552) were prepared following supplier instructions and diluted 1 : 1000 in growth medium. Cells were routinely trypsinized and incubated in the cell-tracker diluted medium for 30 min. Finally, cells were washed twice with 1× PBS to remove the excess of cell tracker before culturing in the devices. CMTPX staining of the donor fibroblasts allowed for a visualization both cell types within the device, distinguishing CMTPX^+^ fibroblasts from the GFP-expressing tumor cells (Fig. 3B).

For immunofluorescence staining, 1000 fibroblasts per well were seeded in a glass-bottom 96 well plate (Greiner, 655086). After 48 h, cells were washed once with PBS and fixed with 4% paraformaldehyde (PFA) (Alfa-Aesar, AA433689M, in PBS) for 15 min. Washing buffer (0.1% PBS-Tween 80) (Sigma-Aldrich, P1754) and blocking buffer (3% bovine serum albumin) (BSA, Sigma-Aldrich, A9056) in 0.1% PBS-Tween 80 were made in advance and stored at 4 °C until use. Unless specified otherwise, steps took place at room temperature. Cells were washed 3 times between every step. Cells were permeabilized for 20 minutes with 0.2% Triton® X-100 (MP Biomedicals, 807426) at and incubated in blocking buffer for 1 h. Thereafter, cells were stained with mouse anti-podoplanin (Abcam, ab10288) overnight at 1 : 100 in blocking buffer. Finally, cells were stained with 488 secondary antibody (anti-mouse, Thermo Fisher A32723, 1 : 1000), phalloidin-TRITC (Sigma, P1951), and DAPI (ThermoFisher, D3571) as a total nuclei indicator in blocking buffer + 10% goat serum (Thermo-Fisher, 31872).

For 3D immunofluorescence staining in devices, cells were washed for 30 min between each step, and the buffers used were the same as for 2D immunofluorescence. Cells were fixed with 4% PFA for 15 min, then incubated for 30 min with 0.2% Triton® X-100 to permeabilize the cells. Finally, devices were incubated with 10 μL of blocking buffer at 4 °C overnight. To evaluate proliferation, cells were stained for the proliferation marker Ki67 (1 : 50 in blocking buffer, ThermoFisher RM9106-S) overnight, and washed for 24 h afterward. Subsequently, we stained with secondary antibody (Thermo-Fisher A-11008).

### ITGA6 silencing

MDA-MB-231 GFP cells were seeded at 250 000 cells per well of a 6-well plate and transfected using Lipofectamine RNAiMAX Reagent (Ref # 13778030). A scrambled control (Silencer Select siRNA Negative Ctl #1, Invitrogen, Ref # 4390843) and 2 commercial ITGA6 Knockdown siRNAs (Silencer Select siRNA ITGA6 Lot A502N7NV (ITGA6 KD #1) and Lot A502N7NW (ITGA6 KD #2), Invitrogen, Ref # 4390824) were used, along with the parental MDA-MB-231 GFP cells, per manufacturer instructions. Media was replaced with fresh complete media 24 h post-transfection. Cells were then trypsinised and seeded in the microdevices as previously described. Integrin alpha-6 knockdown was validated through Western blot at 48 h post-transfection.

### Western blot

Protein concentrations were measured using the Pierce BCA Protein Assay kit (Thermo Scientific, 23225) and 30 μg protein was loaded per well and assessed by total protein staining (MilliporeSigma Fast Green FCF) to ensure equal protein loading. Primary antibody against ITGA6 (Thermo Scientific, CD49F, cat# 710209, 1 : 1000), LN-111 (Novus Biological, NB300-14455, 1 : 1000), and TSP-1 (Abcam, ab267388, 1 : 1000) overnight at 4 °C, followed by horseradish peroxidase-conjugated secondary antibody (Jackson ImmunoResearch Donkey anti-Rabbit, 711-035-152) (1 : 5000) for 1 h at room temperature. Blots were imaged using the LiCor Odyssey Fc Imager and band intensities were analyzed using Image Studio.

### Statistical analysis

All the experiments were repeated at least three times as independent biological replicates. All results are presented as the mean ± standard deviation of the mean. Data were analyzed using GraphPad Prism 10 (GraphPad Software, La Jolla, CA) and statistical significance was set at *p* < 0.05. One-to-one comparisons were performed with an unpaired Student *t*-test with Welch's correction (if SDs were inhomogeneous) after the normal distribution was proved *via* the Shapiro–Wilk test. If the normality test was not passed, a non-parametric test was performed (Mann–Whitney test). Multiple comparisons were performed using two-way ANOVA with Tukey test or a nested one-way ANOVA.

## Author contributions

Conceptualization – MVM, KML, SMP. Methodology – MVM, KML, SAF. Validation – MVM, KML, SAF. Verification – MVM, JRL, SAF, BMB. Formal analysis – MVM, JZ, SAF. Investigation – MVM, SAF, YNV, JRL, KML, BMB, JZ, JMA. Resources – DJB, SMP. Data curation – MVM, SAF, BMB, SMP. Writing, visualization – MVM, SMP. Writing – review & editing, project administration, supervision – MVM, SAF, BMB, JZ, CF, SMP. Funding acquisition – JZ, CF, MVM, SMP, DJB.

## Conflicts of interest

D. J. B. holds equity in Bellbrook Labs LLC, Tasso Inc., Turba LLC, Salus Discovery LLC, Stacks to the Future LLC, Lynx Biosciences Inc., and Onexio Biosystems LLC. J. M. A. is a co-founder of Visynia Biotechnologies LLC. The authors declare no other competing interests.

## Supplementary Material

LC-026-D5LC00711A-s001

LC-026-D5LC00711A-s002

## Data Availability

All data needed to evaluate the conclusions in the paper are present in the paper and/or the supplementary information (SI). Additional raw data related to this paper, including microscopy images and western blots (.tiff files) are stored on the UW-Madison Dryad server for public access (DOI: https://doi.org/10.5061/dryad.1ns1rn98w). Supplementary information is available. See DOI: https://doi.org/10.1039/d5lc00711a.
