# Supplementary material for: Laminin – myCAF interplay promotes α6-integrin-mediated breast cancer cell invasion
Source: Lab Chip. 2026 Aug 21;26(19):5216–31. doi: 10.1039/d5lc00711a (PMC13523129; doi:10.1039/d5lc00711a)
Supplement: Supplementary file 1 [file LC-026-D5LC00711A-s002.pdf]

Supplementary Information (SI) for Lab on a Chip.
This journal is © The Royal Society of Chemistry 2026
Supplementary Information (SI) for Lab on a Chip.
This journal is © The Royal Society of Chemistry

**SUPPLEMENTAL INFORMATION**

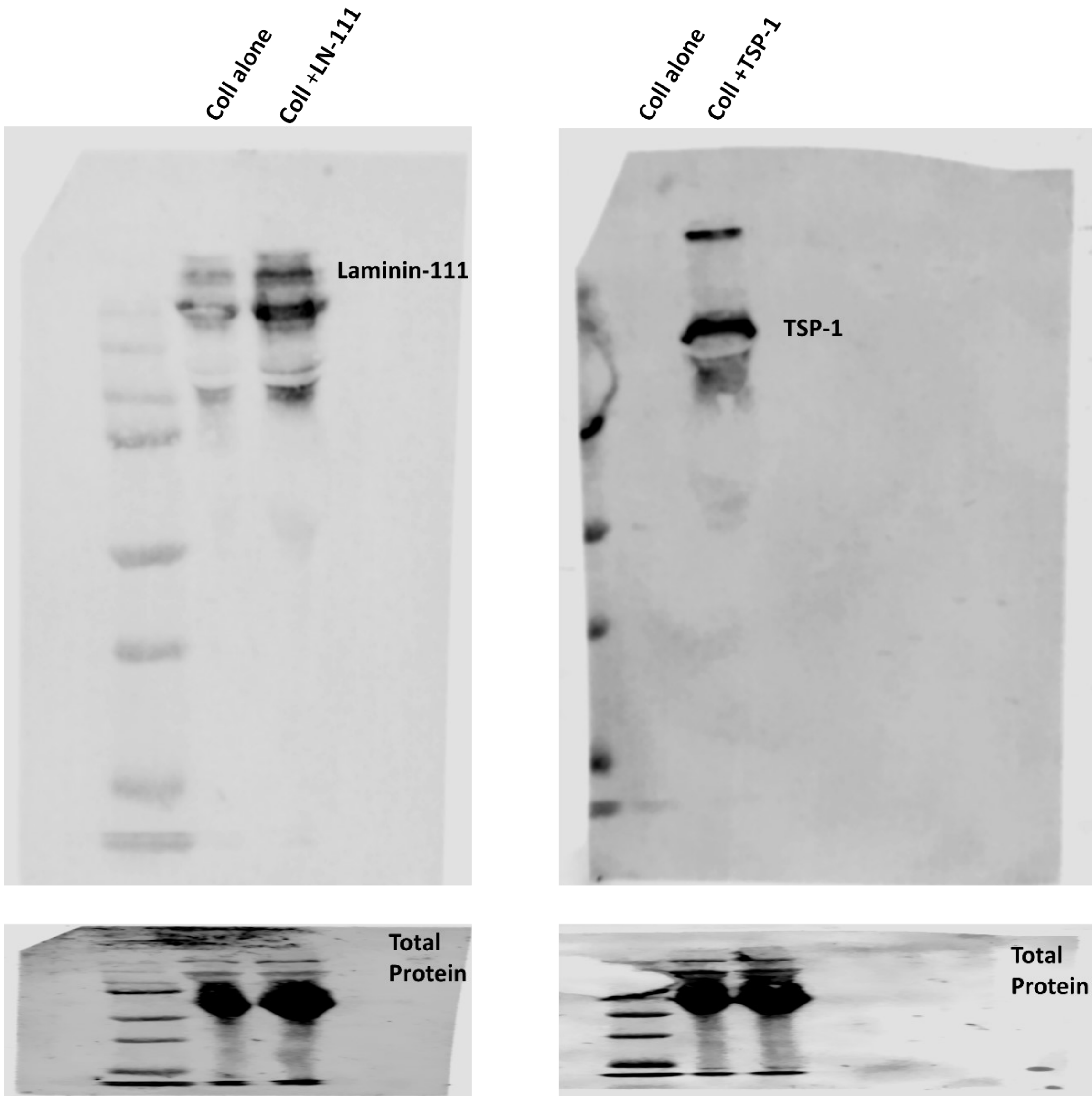


**Figure S1:** Whole gels for western blot and total protein to validate Laminin-111 (LN) and thrombospondin-1 (TSP-1) remain in the hydrogel after the hydrogels have been polymerized and washed. Bands for Laminin-111 (250 KDa) and thrombospondin-1 (180 KDa), can be observed and compared with the molecular weight ladder (BioRad, 1610374).

Figure S2:

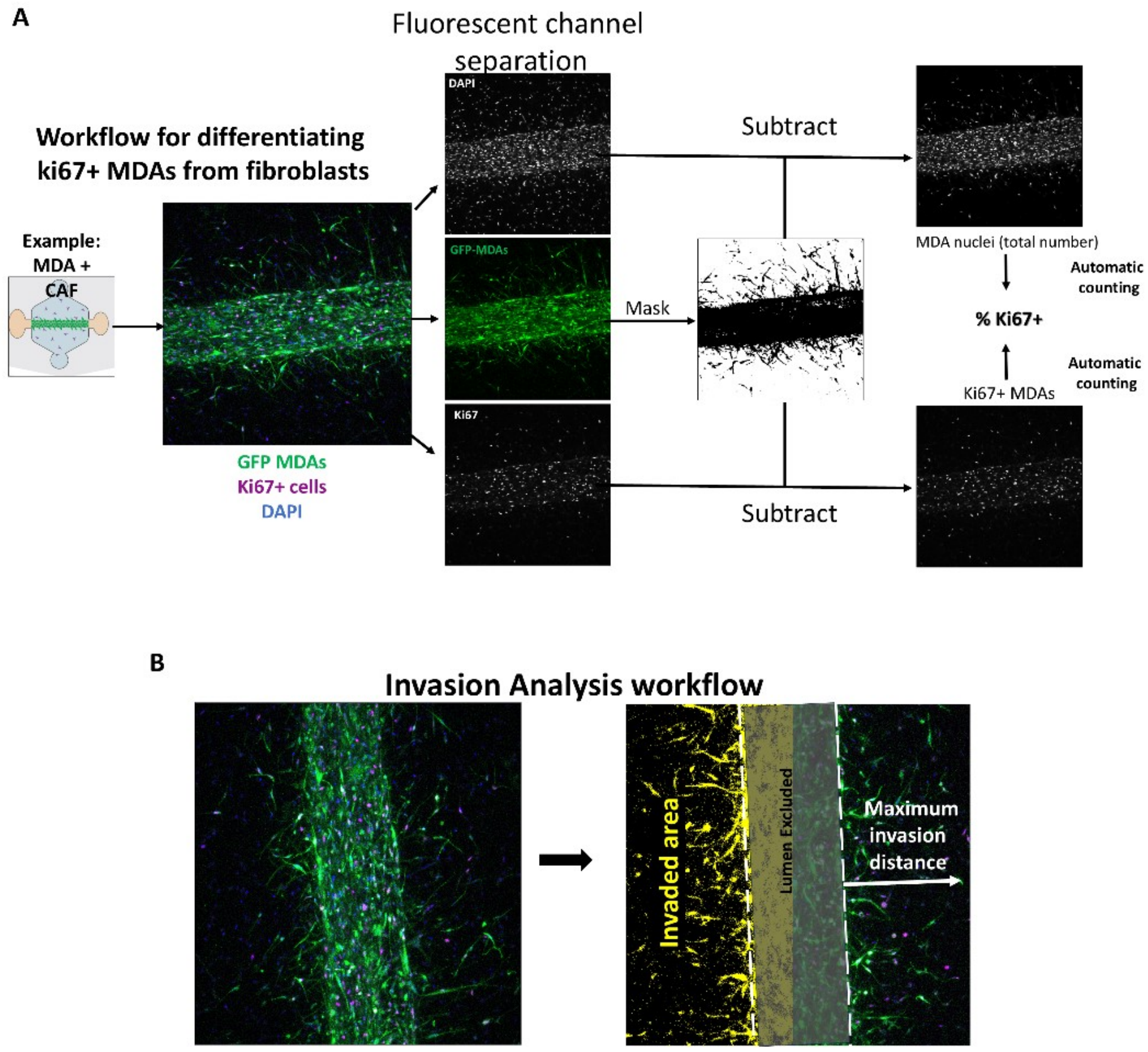


**Supplementary Figure 2:** **A)** Schematic of automated image analysis of Ki67 expression specifically in tumor cells. First the fluorescent channels are separated and a binary mask is generated of the GFP+ tumor cells. Both DAPI+ and Ki67+ cells outside of the mask are subtracted from the respective single channel images. Finally, the percentage of Ki67+ cells / total DAPI+/GFP+ cells is determined. **B)** Schematic of parameters analyzed in the z-projected confocal images of tumor cells after 72 h in co-culture in the IDC invasion model. For invasion analysis, first the lumen is excluded, then to the left of the dashed yellow line (edge of the lumen) a mask of all GFP+ cells is generated to quantify invasion area. The outside the right side of the lumen (white dashed line) a mask of GFP+ cells is generated to quantify the maximum distance of cell invasion into the matrix, with the measurement being performed perpendicular to the edge of the lumen to the farthest cell.

Figure S3:

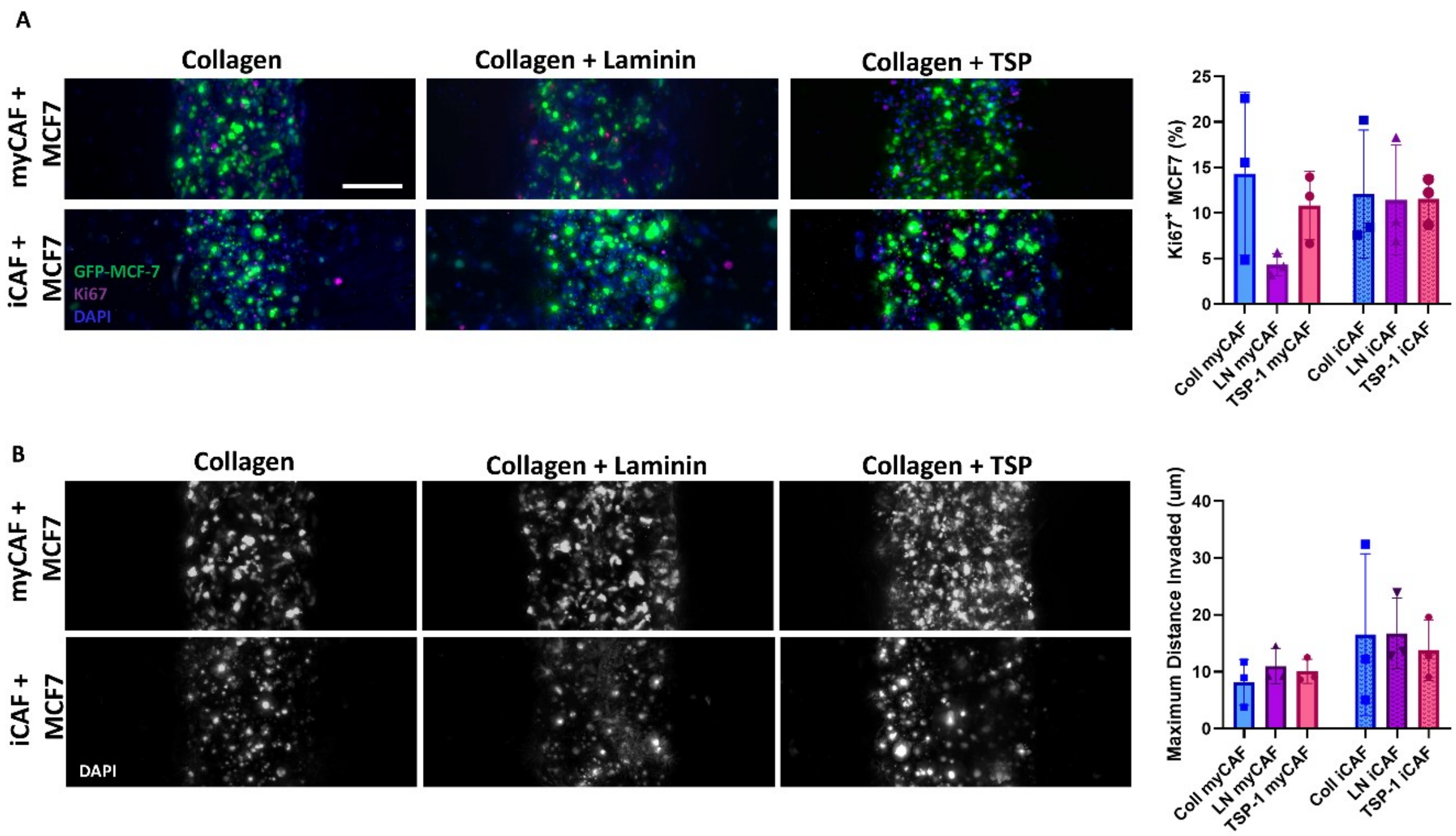


**Supplemental Figure 3: A)** Representative immunofluorescent images of Ki67+ (purple) and GFP+ MCF-7 cells for each matrix condition. Quantification of the percentage of Ki67+ MCF-7 cells showed variable results ranging from ~5 – 20% proliferation, with no statistical differences detected for any of the matrix conditions. B) Representative immunofluorescent images of DAPI+ MCF-7 cells. Very few cells invaded outside of the lumen boundary. Quantification of the maximum invasion distance of invasion resulted in no statistical difference detected for any of the matrix conditions. N=3 cultures per condition with at least 3 technical replicates each. Scale bar = 100 µm
